# Using Ontologies for the Online Recognition of Activities of Daily Living†

**DOI:** 10.3390/s18041202

**Published:** 2018-04-14

**Authors:** Alberto G. Salguero, Macarena Espinilla, Pablo Delatorre, Javier Medina

**Affiliations:** 1 Department of Computer Science, University of Cádiz, Cádiz 11519, Spain; pablo.delatorre@uca.es; 2 Department of Computer Science, University of Jaén, Jaén 23071, Spain; mestevez@ujaen.es (M.E.); jmquero@ujaen.es (J.M.)

**Keywords:** activity recognition, smart environments, ontology, data-driven approaches, knowledge-driven approaches

## Abstract

The recognition of activities of daily living is an important research area of interest in recent years. The process of activity recognition aims to recognize the actions of one or more people in a smart environment, in which a set of sensors has been deployed. Usually, all the events produced during each activity are taken into account to develop the classification models. However, the instant in which an activity started is unknown in a real environment. Therefore, only the most recent events are usually used. In this paper, we use statistics to determine the most appropriate length of that interval for each type of activity. In addition, we use ontologies to automatically generate features that serve as the input for the supervised learning algorithms that produce the classification model. The features are formed by combining the entities in the ontology, such as concepts and properties. The results obtained show a significant increase in the accuracy of the classification models generated with respect to the classical approach, in which only the state of the sensors is taken into account. Moreover, the results obtained in a simulation of a real environment under an event-based segmentation also show an improvement in most activities.

## 1. Introduction

Sensor-based activity recognition [1] is a very relevant process at the core of smart environments. This type of activity recognition is focused on recognizing the actions of one or more inhabitants within the smart environment based on a series of observations of sub-actions and environmental conditions over a period of finite time. It can be deemed as a complex process that involves the following steps: (i) select and deploy the appropriate sensors to be attached to objects within the smart environment; (ii) collect, store and pre-process the sensor-related data and; finally, (iii) classify activities from the sensor data through the use of activity models.

Sensor-based activity recognition is particularly suitable to deal with activities that involve a number of objects within an environment, or instrumental Activities of Daily Living (ADL) [2]. Approaches used for sensor-based activity recognition have been divided into two main categories: Data-Driven Approaches (DDA) and Knowledge-Driven Approaches (KDA) approaches [1].

The former, DDA, are based on machine learning techniques in which a preexistent dataset of user behaviors is required. A training process is carried out, usually, to build an activity model, which is followed by a testing process to evaluate the generalization of the model in classifying unseen activities [3]. The advantages of the DDA are the capabilities of handling uncertainty and temporal information. However, these approaches require large datasets for training and learning and suffer from the data scarcity or the cold start problem.

With KDA, an activity model is built through the incorporation of rich prior domain knowledge gleaned from the application domain, using knowledge engineering and knowledge management techniques [4,5]. KDA has the advantages of being semantically clear, logically elegant and easy to get started. Nonetheless, they are weak in dealing with uncertainty and temporal information, as well as the activity models can be considered as static and incomplete.

In the context of KDAs, ontologies for activity recognition have provided successful results. Ontologies can be described as structured vocabularies that explain the relations among their terms (or classes). They are formed by concepts and relations that can be combined to form more complex class expressions. In this kind of approach, interpretable activity models are built in order to match different object names with a term in an ontology that is related to a particular activity. Activity models are usually modeled in this case as class expressions that describe the events that must necessarily occur for each kind of activity. Thanks to the high formalization of ontologies, automatic reasoning mechanisms can be used to infer information that has not been given explicitly and thus improve the activity classification models.

Some hybrid approaches have been developed [6,7] that take advantage of the main benefits provided by DDA and the use of an ontology. Thereby, ontological ADL models capture and encode rich domain knowledge and heuristics in a machine-understandable and -processable way. In this work, we propose a hybrid approach for the online activity recognition. The main contributions of this paper are:

- Automatic generation of features for DDA: The dataset has been described in the form of an ontology. The relevant concepts and properties in the ontology are combined and transformed to generate new concepts, which are then evaluated to determine their relevance. This process is repeated until a certain number of concepts describing the activities of the dataset are obtained. All the new concepts are then taken as input features by the supervised learning algorithms to generate the activity classification models. These concepts also provide knowledge to describe the activities under a richer and interpretable representation.
- An ontology for the mining of ADL: One of the main problems of ontologies is their low performance. We describe in this work an ontology that has been specifically developed for the mining of ADL. This ontology greatly reduces the amount of resources needed for reasoners and improves the efficiency of the whole process.
- Online activity recognition: In this paper, we extend our previous work [8] and propose an approach for the online recognition of activities. In that work, we proposed a hybrid approach for the recognition of activities after a pre-segmentation process in which the sensor data stream was divided into segments, also known as temporal windows, by using the begin and the end labels of each activity [9,10]. The begin label indicates the starting time of the activity, and the end label indicates the ending time of the activity. Therefore, each segment of the sensor data stream exactly corresponds to an activity. The DDA approach offers excellent results for offline activity recognition where the begin and the end labels of each activity in the dataset are known. However, the successful results that DDA provides cannot be transferred to online activity recognition, since the begin label cannot be predicted [9,11]. In this paper, we propose a mechanism to dynamically calculate the size of temporal windows for each type of activity.

An evaluation of the approach proposed in this work is undertaken with a popular dataset. The results obtained with our proposal are analyzed and compared with respect to those obtained using a classic approach, in which only the status of the sensors is taken into account. The results show significant improvements in the performance for the classification models based on our proposal.

The remainder of the paper is structured as follows: In Section 2, we introduce some notions about ontologies that are needed to understand our proposal and review some related works in the literature. Section 3 proposes the methodology that generates features from the dataset, once converted to an ontology. Section 4 presents an empirical study in which we analyze the results obtained by our proposal for a well-known dataset. Finally, in Section 5, conclusions and future work are presented.

## 2. Background

In this section, we first review some relevant concepts related to ontologies that are needed in order to understand our proposed methodology. Then, some related works are also reviewed.

### 2.1. Ontologies

Ontologies are used to provide structured vocabularies that explain the relations among terms, allowing an unambiguous interpretation of their meaning. Ontologies are formed by concepts (or classes), which are, usually, organized into hierarchies [12,13], the ontologies being more complex than taxonomies because they not only consider type-of relations, but they also consider other relations, including part-of or domain-specific relations [14].

In an ontology, the symbol ⊤ stands for the *top* concept of the hierarchy, all other concepts being subsets of ⊤. The *subsumption* relation is usually expressed using the symbol A⊑B, meaning that the concept *A* is a subset of the concept *B*. Concepts can also be specified as logical combinations of other concepts.

The semantics of operators for combining concepts is shown in Table 1, where $C,C_{1},C_{2}⊑⊤$, *R* is a relation among concepts, $\Delta_{I}$ is the domain of individuals in the model and *I* is an interpretation function.

An ontology expresses what individuals, also called objects, belong to which concepts. Moreover, it is possible to declare properties to relate individuals, organizing them into a hierarchy of sub-properties and providing domains and ranges for them. Usually, the domains of properties are concepts, and ranges are either concepts or data types. A declared property can be defined as transitive, symmetric, functional or the inverse of another property (R${}^{-}$).

The main advantage of ontologies is that they codify knowledge and make it reusable by people, databases and applications that need to share information [14,15]. Due to this, the construction, the integration and the evolution of ontologies have been critical for the Semantic Web [16,17,18]. However, obtaining a high quality ontology largely depends on the availability of well-defined semantics and powerful reasoning tools.

Regarding Semantic Web, a formal language is OWL [19,20], which is developed by the World World Wide Web Consortium (W3C): Originally, OWL was designed to represent information about categories of objects and how they are related. OWL inherits characteristics from several representation languages families, including Description Logic (DL) and Frames basically. OWL is built on top of the Resource Description Framework (RDF) and RDF Schema (RDFS). RDF is a data-model for describing resources and relations between them. RDFS describes how to use RDF to describe application and domain-specific vocabularies. It extends the definition for some of the elements of RDF to allow the typing of properties (domain and range) and the creation of subconcepts and subproperties. The major extension over RDFS is that OWL has the ability to impose restrictions on properties for certain classes.

### 2.2. Related Works

We can find in the literature many works dealing with different aspects of ADL recognition. Within the field of activity recognition, we can distinguish between two main areas of research: activity segmentation and activity classification. Works in the former group try to determine the exact period of time during which the activities are actually taking place [21,22]. The proposals of the latter groups are focused on determining the specific type of activity that people are carrying out, building a feature representation from the sequence of sensor events both in online approaches [9,23], as well as offline classification from labeled activities [24]. Usually, both types of approaches are based on the division of the data stream into segments, also called windows or time-slots. Three common segmentation approaches can be found in the literature:

- Based on activity: This is a popular segmentation approach, also called explicit, being adopted by a wide range of offline DDA proposals for sensor-based activity recognition because of its excellent results [25]. Typically, the sensor data stream is divided into segments coinciding with the starting and ending point of time for each activity, whose activation within them provides a straightforward feature representation from binary sensors [24]. The main disadvantage of this approach is that it is not feasible for online activity recognition because it is not possible to know when the activities are going to start or end.
- Based on time: In this approach, the sensor data stream is divided into segments of a given duration [26]. The main problem of this approach is identifying the optimal length of the segments, since it is critical for the performance of activity recognition [27]. Initial approaches proposed a fixed time segmentation for evaluating the activation of binary sensor within temporal windows. These approaches usually employed windows of 60 s in length, which provide good performance in daily human activities recognition [9,23]. Recent works propose the use of more elaborated methods to identify the optimal size of windows per activity by using statistical analysis: (i) the average length of the activities and the sampling frequency of the sensors [28]; or (ii) a weighted average by the standard deviation of the activities [22]. This approach has been also used in activity recognition based on wearable sensor devices [29].
- Based on events: Another approach consists of dividing the sensor data stream based on the changes in sensor events [30]. The main problem with this approach is separating sensor events, which may correspond to different activities that can be included in the same segment. On the one hand, this approach is adopted by some research works that analyze sensors providing continuous data from wearable devices, such as accelerometers [10]. On the other hand, the segmentation based on events in binary sensors has been proposed to evaluate activity recognition together with dynamic windowing approaches [21].
- Others: Others works include ad hoc segmentation approaches, such as [31] or [32], where a semantic-based segmentation approach for the online sensor streams is proposed.

In our proposal, we include a mixed approach. First, a segmentation based on time is adopted to define the relevant events under a sliding temporal window [28] for each activity. The optimal size of the sliding temporal window is adapted by a statistical analysis [22]. Initially, the performance of this time-based segmentation is evaluated at the end of each activity. Secondly, to evaluate our approach under real circumstances, we have performed another experiment in which the segmentation based on events has been used. In this experiment, we try to determine the activity being performed each time a sensor changes its state [21]. The use of the explicit segmentation based on activity has been discarded because it cannot be used for online activity recognition in a real situation.

Identifying a suitable sensor-based representation for building feature vectors is also an important key factor in the recognition of ADL [26]. Previous works have been focused on evaluating expert-defined representations of binary sensors, such as raw activation, last activation or change point [9,33]. Nevertheless, these representations based on human interpretation hide more complex relationships among sensors. Instead, the proposal presented in this paper automatically generates multiple class expressions, which represent different states of the sensors and sequences of events during the activity. The supervised learning algorithms determine the best representation through a feature selection process. Following, we review some works that also use structured knowledge sources to generate new features for classifying tasks.

A framework that generates new features for a movie recommendation dataset is proposed in [34]. They use that framework to construct semantics features from YAGO, a general purpose knowledge base that was automatically constructed from Wikipedia, WordNet and other semi-structured web sources. Then, they manually define a set of static queries in SPARQL language that are used to add information to the original dataset, such as its budget, release date, cast, genres, box-office information, etc. Despite being a proposal that is very similar to ours, it is important to note that the set of features in our case is fully automatically generated, without the need for human interaction.

The authors in [35] also propose the use of ontologies to generate new features. They expand features from the original feature in a breadth first search manner considering the rules for semantically-correct paths defined by Hirst and St-Onge [36]. Only concepts on outgoing paths from the original entity conforming to these patterns are considered as possible features in the further process. Although they plan to test their proposal with two ontologies, they are actually dealing with the underlying RDF graph of those ontologies. They do not make use of the inference mechanisms of ontologies, nor the formal logic behind them. They just use the user-defined relationships between concepts in the RDF graph in order to relate the original concept to the concepts in its context.

Paulheim [37] also proposes another technique that employs user-defined relations between concepts in the RDF graph of ontologies for the automatic generation of features. Its main goal is the generation of possible interpretations for statistics using Linked Open Data. The prototype implementation can import arbitrary statistics files and uses DBpedia to generate attributes in a fully-automatic fashion. Furthermore, the author argues that their approach works with any arbitrary SPARQL endpoint providing Linked Open Data. The use of the inference mechanisms of ontologies is also very limited in this work.

The main difference with respect to our proposal is that all previous works propose the use of external knowledge to generate new features, whereas we only consider the information in the original dataset to do so. There are other proposals that also generate new features by just using the data in the dataset. The Latent Dirichlet Allocation (LDA) model is proposed in [38] to capture the correlation between features and activities by using an offline approach, where both the start and the end labels of the activities are known. Then, they apply the learned classification model for the online recognition of activities. They perform this step by constructing a fixed dimensional feature vector, which is actually composed of two groups of features. One group identifies the basic features in the sliding window, such as the day, the time of the first and the last sensor events, the temporal span of the window, the sensors of the first and the last events and the previous activity. The rest of the features correspond to estimated probability distributions of activities and sensors events. The features are thus generated by using a predefined set of ad hoc rules, the dimension of the feature vector always being the same. In contrast, our proposal uses ontologies to automatically generate features, the final dimension of the feature vector being decided by the user.

We can also find in the literature many ontologies for the description of ADLs [5,39,40,41]. However, most of them have been designed to be as expressive as possible, defining a huge amount of classes and properties. This makes it more difficult for our methodology to find relevant class expressions, since the search space grows exponentially. The ontology proposed in [40], for example, contains one hundred and sixty-five predefined activities and thirty-three different types of predefined events (“atomic activities”). It also incorporates other types of entities that are specific to the dataset, such as the person performing the activity and his or her posture. Furthermore, all properties are flat, i.e., without any characteristic (inverse, functional, etc.), because the reasoning about the order of events is done in an external rules system. The same applies to the ontology proposed in [5], where there are properties to associate a sensor with the object in which it is located and even its manufacturer. Their authors distinguish between simple and compound activities, and they are organized into hierarchies. There are also dozens of activities and properties already predefined in the ontology that do not correspond to the activities in the datasets of the experiment described in Section 4.2. The ontology proposed in [41] is oriented towards the development of an ADL monitoring system that can interact with the users through mobile networks. It includes concepts and properties to implement a message service between the user and the monitoring system.

The ontology proposed in [42] is the most similar to the one used in the experiment of this work, described in a later section. It defines some concepts that are not needed in the experiment, such as the type of the sensor or its location. However, the main problem with this ontology is its inefficiency. It has been designed for KDA approaches, and it encodes some complex class expressions that take much time to compute. Even if those concepts are removed (they are defined for a specific dataset), an exploratory analysis shows that the time required to determine the instances of some of its concepts ranges from seven minutes to ten hours, whereas the entire process takes no more than a few seconds, in most cases, when using the proposed ontology.

The system proposed in [31] requires human intervention. This includes initial inputs of domain knowledge, manual specification of the seed ontological activity models and human validation and update of learned activities at the end of each single iteration. The quality of the results depends on the quality of the rules created ad hoc for the dataset. Only some phases are completely automatic. They employ a small, private dataset, although they plan to test their approach using some publicly available activity datasets.

The methodology proposed in this paper also shares some characteristics with Class Expression Learning (CEL) techniques. The algorithms for CEL are mainly used in the field of ontology engineering [43]. They can be used to suggest new class descriptions that are relevant to the problem while the ontologies are being developed. The objective of CEL algorithms is to determine new class descriptions for concepts that may be used to classify individuals in an ontology according to some criterion. More formally, given a class *C*, the goal of CEL algorithms is to determine a class description *A* such that A=C. Given a set of positive and negative examples of individuals in an ontology, the learning problem consists of finding a new class expression or concept such that most of the positive examples are instances of that concept, whereas the negative instances are not.

The main difference with respect to our proposal is that the result of CEL algorithms is always a DL class expression, whereas the result of the proposed methodology is a set of DL class expressions, which do not always describe positive instances. Sometimes, the features of negative instances provide valuable information to the classification model. In our case, the entire set of generated class expressions is treated as features. The classifier may combine the DL class expressions as necessary, without the need to produce the result in the form of logical axioms that describe positive instances. This is the reason why the classifiers based on our proposal perform better than the CEL algorithms. In addition, the rigidity of DL makes the classification model less tolerant to conflicting and incoherent situations due to faulty sensing hardware or communication problems [44].

## 3. Methodology

This section describes the proposed methodology, which is made up of several independent applications. The purpose of the methodology is to add relevant features to the dataset to improve the accuracy of the classifiers in ADL by means of ontologies.

### 3.1. An Ontology for the Description of Activities

To describe the activities in [23], an ontology has been developed in OWL (http://agsh.net/adl/1.0/adl.owl). The ontology defines two basic concepts, *Activity* and *Event*, which respectively represent all the activities in the dataset and the activation of the sensors during these activities. Fourteen new subclasses of the *Event* class have been defined, each of them representing the activation during the activity of each of the sensors in the dataset (for simplicity, only the activation of the sensors has been taken into account for the experiment in Section 4.2). However, it is also possible to consider the deactivations of the sensors by just enabling a flag in the application developed for loading the dataset in the ontology. The class *Freezer_set*, for example, represents the set of events corresponding to the activation of the sensor in the freezer.

**Figure 1:**
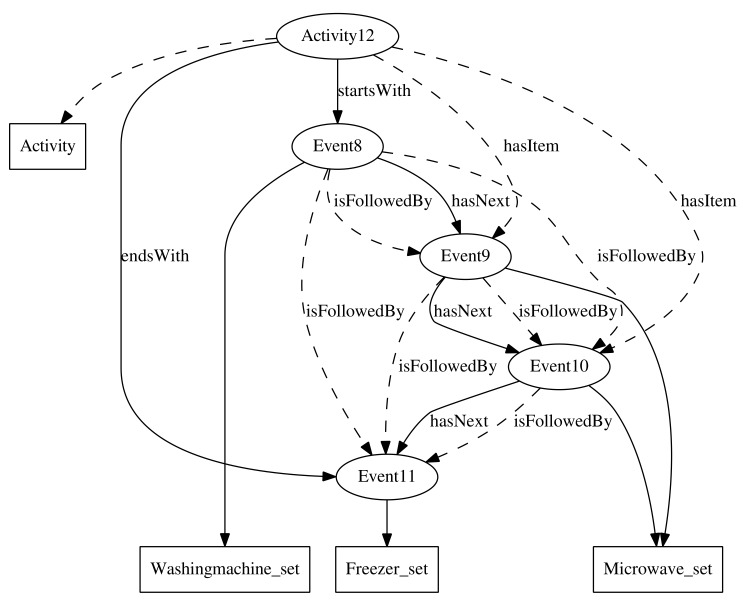
Ontology example.

To relate sensors events to the activities, we have defined four properties, shown in Figure 1. The properties *startsWith* and *endsWith* relate a particular activity to the first and the last events that occur during that activity, respectively. Both properties have been defined as functional, since an activity can only begin and end with a unique event. They have been declared as sub-properties of the *hasItem* property, which relates an activity to the events that have occurred during that activity. The property *hasItem* has been defined as an inverse functional, since an event may occur just in one activity. The class description *hasItem some Freezer_set*, for example, represents all those activities during which the freezer sensor has been fired.

The order among events produced in an activity is maintained through the *hasNext* property. This property has been defined as functional and inverse functional, since an event can only be immediately followed or preceded by a single event. It has also been declared as an asymmetric and irreflexive property, since an event that happens after another event cannot happen before the former one, nor after itself. This property allows us to describe activities as chains of events. The class description *startsWith some (Washingmachine_set and hasNext some (Microwave_set and hasNext some Microwave_set))*, for example, represents the activities that begin with the activation of the sensor of the washing machine, which is immediately followed by two consecutive activations of the microwave sensor. The activity *#Activity12* in Figure 1 is an example of activity described by the above class description.

The *hasNext* property has been declared as a subproperty of the *isFollowedBy* transitive property, which relates an event to all events that happen after it in an activity. Events related through the latter property do not have to occur consecutively in the activity. The class description *hasItem some (Washingmachine_set and isFollowedBy some Freezer_set)* is another way of describing the activity *#Activity12* of the example in Figure 1.

Due to the high formalization of ontologies, it is not necessary to make all relations in the dataset explicit. Many of them may be inferred by the reasoner. Knowing that *#Event8 hasNext #Event9*, the reasoner may infer that *#Event8 isFollowedBy #Event9*, since hasNext⊑isFollowedBy. In addition, if *#Event9 hasNext #Event10*, the reasoner may easily infer that *#Event8 isFollowedBy #Event10*, since the property *isFollowedBy* has been declared as transitive.

### 3.2. Extended Features Generation

In this section, we explain how the features are generated from the information in the ontology. Basically, the idea consists of the combination of the entities in the ontology (concepts and relations), by means of logic operators, to generate new class descriptions that may be useful for the classification of the activities. Eventually, a class description that describes certain kinds of activities may be found and selected as a new feature for the classifiers. The process is repeated until a sufficient number of new relevant features is found. All the components of the methodology proposed in this work and how the information flows among them are explained in this section.

**Figure 2:**
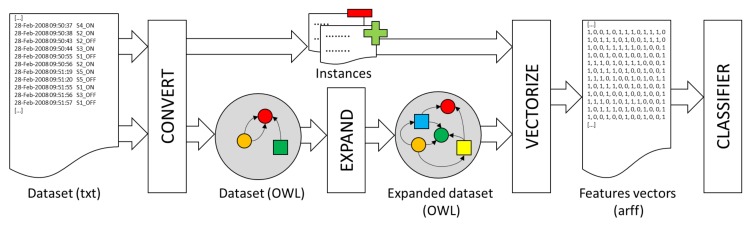
Functional architecture.

The system starts from a dataset with a set of labeled activities (see Figure 2). First of all, it is necessary to convert the dataset information into an ontology. In the experiment described in Section 4.2, an application has been developed to convert the information in [23] into an ontology following the rules in Section 3.1. In this first step, two text files are also generated that contain: (a) the list of individuals in the ontology of the kind of activity to be recognized by the classifier (positives); and (b) the rest of the individuals (negatives). These lists of individuals will be used to generate the input data for the classifier in a later step.

Next, it is necessary to expand the definition of the *Activity* concept in the ontology. The expansion process consists of generating new class descriptions that represent different patterns of activities, without taking into account the specific type of activity that is going to be recognized by the classifier. More specifically, the *OWLExpand* (all the software is freely available under the terms and conditions of the GNU General Public License at https://sourceforge.net/p/owlmachinelearning/) program takes the concept to be expanded (*Activity*) as an argument and uses a given set of classes, properties and operators to construct new class descriptions in DL. All the concepts in the ontology that at least describe some individual in the ontology have been taken as the set of classes *L*. All properties defined in the ontology have been taken as the set of properties *P*. The set of operators *O* is specified by the user and consists of a subset of all operators that can be used to combine class descriptions (see Section 2.1).

The expansion process begins by combining all the concepts in *L* by means of *O* operators. The complement operator (C) results in class expressions of the form $not\;c_{i}$, where $c_{i}\in L$. Class expressions such as *not Cupboard_set* or *not Activity* are produced using the complement operator, for example.

All class descriptions in *L* are combined with themselves in the case of operators that require two class descriptions, resulting in expressions of the form $c_{i}\;o_{k}\;c_{j}$, where $c_{i},c_{j}\in L$, i≠j and $o_{k}\in \left\{and,or\right\}$. In this process, expressions such as *Event and Cupboard_set* or *Cupboard_set or HallBedroom_Door_set* are generated, for example.

There are operators that require a property to form valid class descriptions. They are the existential quantifiers and the universal quantifier. In this case, the expansion process combines all class descriptions in *L* with all properties in the ontology, producing expressions of the form $p_{i}\;o_{k}\;c_{j}$, where $p_{i}\in P$, $o_{k}\in \left\{some,all\right\}$ and $c_{j}\in L$. *startsWith some HallBedroom_Door_set* or *isFollowedBy all Cupboard_set* are examples of expressions generated by existential and universal quantifiers. These class expressions represent all those individuals that begin with the firing of the HallBedroom_Doorsensor and all those individuals that are only followed by Cupboardsensor activations, respectively.

The last type of operator that implements the *OWLExpand* application is the cardinality constraints. These operators limit the number of individuals to which an individual may be related among a given property. The class descriptions generated with these operators have the form $p_{i}\;or_{k}\;n\;c_{j}$, where $p_{i}\in P$, $o_{k}\in \left\{min,max,exact\right\}$, $c_{j}\in L$ and n∈N. Expressions such as *isFollowedBy min 4 Event* or *isFollowedBy exact 2 Cupboard_set* are generated, for example, representing the set of individuals followed by at least four sensor activations and the set of individuals followed by exactly two activations of the *Cupboard* sensor, respectively. The number of constraints to be generated is virtually infinite since n∈N. It is the user who must specify the values for *n*. For example, n∈{2,3} in the experiment of Section 4.2.

All the expressions generated are added to *L*, and the process is repeated again. However, not all of the expressions generated are relevant. Some of them are simply unsatisfiable. A class expression such as *hasItem some Activity*, for example, is unsatisfiable since the range of the property *hasItem* is the *Event* concept, which is defined to be disjoint with the concept *Activity*. There cannot be an individual in the ontology that meets such a restriction. For the same reason, expressions like *hasItem some startsWith some HallBedroom_Door_set* are also unsatisfiable, since the domain of the *startsWith* property is the concept *Activity*. Only satisfiable class expressions are added to *L*.

On the other hand, not all class expressions in *L* describe activities. With the help of the reasoner, a new set V⊆L is created, which contains all the class expressions in *L* that describe activities. These are the expressions that the program *OWLExpand* produces as a result, in a text file.

The *OWLVectorize* application takes the class expressions generated in the previous step as input and produces a table with *k* rows and *n* binary columns, where *k* is the number of annotated activities in the dataset and *n* is the number of class descriptions generated in the expansion process. Each of the rows is therefore a vector $F^{k}=\left\{f_{1}^{k},\ldots ,f_{j}^{k},\ldots ,f_{n}^{k},f_{n+1}^{k}\right\}$. Each of the *n* generated class expressions corresponds to a feature $f_{j}^{k}\in F^{k}$. $F_{j}^{k}=1$ if the activity *k* is an instance of the class description *j*. $F_{j}^{k}=0$, otherwise. $F_{n+1}^{k}=1$ if the activity *k* is an instance of the kind of activity to be recognized by the classifier (positive). $F_{n+1}^{k}=0$, otherwise. The list of annotated individuals generated at the beginning of the process is used for this purpose. An example of the results obtained by this application is shown in Table 2.

## 4. Experiment

In this contribution, a popular activity recognition dataset [23] of a smart environment is used to evaluate the performance of our proposal. In this section, we first describe the dataset and the experiment. Then, we compare the results obtained by classifiers using the classical approach and classifiers using the methodology proposed in this work.

### 4.1. From the Sensor Data Stream to Feature Vectors

The dataset [23] used in the experiment to evaluate our proposal is composed of binary temporal data from a number of sensors, which monitored the ADLs carried out in a home setting by a single inhabitant. This dataset was collected in the house of a 26-year-old male who lived alone in a three-room apartment. This dataset contains 245 activities that are annotated in the stream of state-change sensors generated by 14 binary sensors.

Each sensor is located in one of 14 different places within a home setting: microwave, hall-toilet door, hall-bathroom door, cup cupboard, fridge, plate cupboard, front door, dishwasher, toilet flush, freezer, pan cupboard, washing machine, grocery cupboard and hall-bedroom door. Sensors were left unattended, collecting data for 28 days in the apartment. Activities were annotated by the subject himself using a Bluetooth headset.

Seven different activities are annotated, namely: going to bed, using the toilet, preparing breakfast, preparing dinner, getting a drink, taking a shower and leaving the house. Table 3 shows the number of instances per activity in the dataset, as well as their mean duration and standard deviation, in seconds. Table 4 shows the number of sensor events per activity.

The set of activities are denoted by $A=\{a_{1},\ldots ,a_{i},\ldots ,a_{N_{A}}\}$, $N_{A}$ being the number of the different types of activities in the dataset. Usually, to build the feature vectors from the activities dataset, the sensor data stream is discretized into a set of *k* temporal windows $W=\{w^{1},\ldots ,w^{m},\ldots ,w^{k}\}$. For simplicity, a single temporal window $w^{m}$ has been created in the experiment for each activity instance. To implement an online recognition of activities, we have made the end of each temporal window $w^{m}$ to coincide with the end of each activity instance. The size of the temporal windows depends on the average duration, $\bar{\Delta_{a_{i}}}$, and the standard deviation, $\sigma_{a_{i}}$, of each type of activity, and we have calculated it according to the expression:

(1) $$\Delta_{w^{m}}=\bar{\Delta_{a_{i}}}+c\cdot \sigma_{a_{i}}$$

where $a_{i}$ is the type of activity of the instance that ends at the same instant as the temporal window $w^{m}$ does and *c* is a factor used to weigh the importance of the standard deviation in the window size.

**Figure 3:**
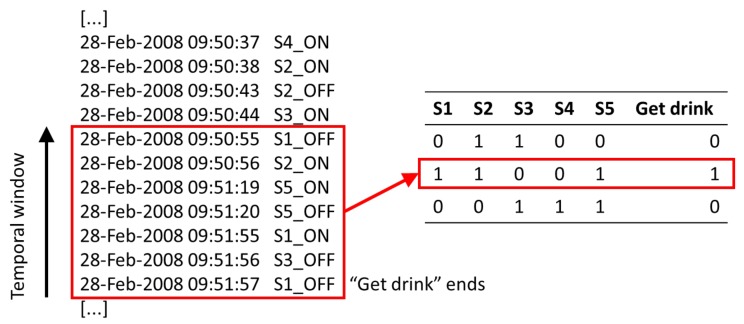
Example of a feature vector for a temporal window computed from the sensor data stream.

The set of sensors in the dataset is denoted by $S=\{s_{1},\ldots ,s_{j},\ldots ,s_{N_{S}}\}$. In the classic approach, each feature vector $F^{k}=\left\{f_{1}^{k},\ldots ,f_{j}^{k},\ldots ,f_{N_{S}}^{k},f_{N_{S}+1}^{k}\right\}$ has $N_{S}+1$ components, $N_{S}$ being the number of sensors. The first $f_{j}^{k};j=\left\{1,\ldots ,N_{S}\right\}$ components are binary values that indicate if the sensor $s_{i}$ was fired at least once, 1, or was not fired, 0, in the temporal window $w^{k}$. The last component $f_{N_{S}+1}^{k}$, also binary, indicates if the temporal window $w^{k}$ represents an instance of the current target activity, 1, or not, 0. Figure 3 shows an example of how a feature vector has been computed from a temporal window.

### 4.2. Experiment Description

In order to evaluate the quality of the methodology proposed in this work, an experiment has been carried out with the activities in [23]. The objective of the experiment is to determine whether or not the target activity has been performed.

The results obtained by four supervised learning algorithms that use a classic DDA to solve this problem have been taken as the reference to measure the efficiency of our proposal. For this purpose, an application that identifies the sensors that have been fired during each of the temporal windows has been developed. The average duration of activities has been used to compute the length of the temporal windows (c=0). The application generates a file in Weka format, following the structure presented in Section 4.1. This file contains an instance for each temporal window and as many features as sensors in the dataset. All the features are binary and specify if the sensor has been fired during the temporal window or not. Finally, it includes a class attribute, also binary, that indicates if the temporal window is an instance of the target activity. Each experiment consists of determining which combination of sensors is fired for a particular activity, such as “take a shower”, for example.

By using the Weka data mining software, we have generated the C4.5 (the Weka implementation of the C4.5 classifier is called J48), Sequential Minimal Optimization (SMO), Voted Perceptron (VP), Random Forests (RF) and Decision Table (DT) classifiers for all the activities in the dataset. Default parameters have been used for all of them. The *Percent correct* value is the default measure provided by Weka to measure the performance of classifiers. It represents the fraction of both the positive and negative activities that are correctly classified, and it is formally defined as:

Percentcorrect=100·(tp+tn)/(tp+tn+fp+fn)

where tp, tn, fp and fn are respectively the true positive, true negative, false positive and false negative instances. The *F-measure* is also often used to measure the accuracy of tests and is defined as:

$$F-measure=2\cdot \frac{precision\cdot recall}{precision+recall}$$

where precision=tp/(tp+fp) and recall=tp/(tp+fn). We have employed both the *Percent correct* and the *F-measure* to measure the performance of all the classifiers generated in the experiment. In the remainder of the paper, the term accuracy will refer to the *F-measure*.

The three most difficult activities to recognize are “go to bed”, “prepare dinner” and “’use the toilet”, as shown in Table 5. These activities have been used to test the performance of our proposal because there is more room for improvement. Although most classifiers correctly classify above ninety percent of the activities, it is worth mentioning that they achieve really low accuracy for some activities, such as “go to bed”. This is mainly due to an imbalance in the dataset. The accuracy achieved in the case of the activity “go to bed” is almost null for most of the supervised learning algorithms, meaning that few or none of the positive instances have been found by them. Actually, a more detailed analysis of the results shows that they classify most of the instances as negative. Because most of the instances in the dataset are negative, they achieve a high percentage of correctly-classified instances. In fact, a majority classifier, where all the instances are classified as negative, would obtain a value of 90.2% for the activity “go to bed”, for example.

The dataset has been transformed to an ontology following the scheme described in Section 3.1. The *OWLExpand* program has been used then to generate new class descriptions describing the *Activity* concept. In order to analyze the relevance of candidate operators for activity recognition, we have used three subsets of DL operators to generate three different sets of new class descriptions. For the first one, all available operators (ACIXMSU) have been used. Only the existential quantifier (S) has been used for the second set. A quantifier is needed in order combine concepts through properties. We decided to use the existential quantifier because the universal quantifier produces very restrictive class expressions. The complement, minimum cardinality and the existential quantifier operators (CSM) have been used for the third set, as an intermediate solution. Moreover, with the aim of analyzing the impact of the number of class expressions being generated, we have created versions with n={20,40,60,80,100,120,140,160,180,200} expressions for each subset of operators.

All files with new class descriptions are evaluated by the *OWLVectorize* application, and a new file in Weka format is generated for each of them. The same five types of classifiers that were employed to evaluate the performance of the classifiers using the classic approach have been used to evaluate the accuracy of the classifiers based on the new approach. Results are discussed in the next section.

To determine the impact of the size of the temporal window on the results, we repeated the experiment for three different values for the *c* factor in Equation (1). The values used were 0, 0.25 and 0.5.

### 4.3. Results

The performance achieved by all the classifiers generated in the experiment for the activities “go to bed”, “use the toilet” and “prepare dinner” is shown in Table 6, Table 7 and Table 8, respectively. For the sake of clarity, only results for n≤100 are shown. No significant values are found for n>100. The first row shows the results obtained by using the classic approach, in which there is a feature for each sensor in the dataset. For the remaining rows, the first column indicates the set of DL operators used to generate the features. The number of features is shown in the second column. The remaining columns indicate the percentage of activities that each supervised learning algorithm has correctly classified for the given number of input features, as well as its *F-measure*. The eighth and the last columns show the best values obtained among all algorithms for each number of features and DL operators sets. The best overall values obtained for each set of DL operators are in bold. Values that are significantly different from the result obtained using the same algorithm with the classic approach are in italics. We have employed the *Paired T-Tester* test of Weka for a confidence of p=0.05 (two tailed). The *corrected* version of the tester has been used because we are using cross-validations in the experiments.

**Figure 4:**
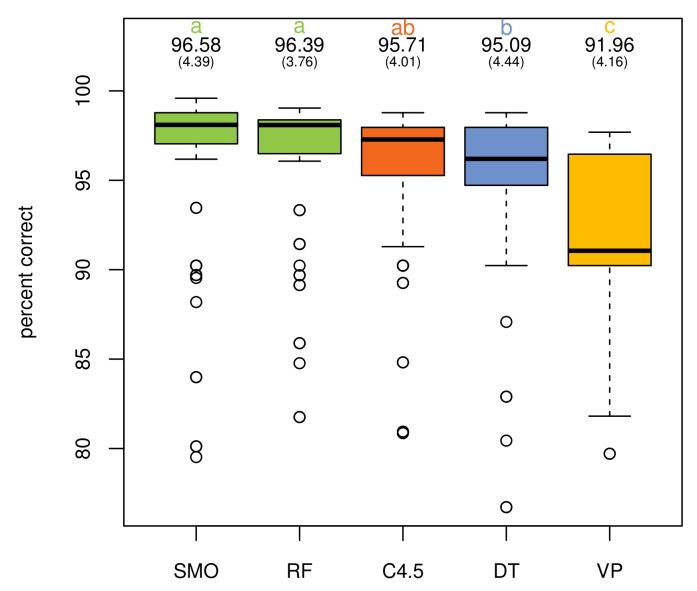
Classifiers’ global performance.

Additionally, a multiple analysis of variance (MANOVA) was conducted to provide evidence about the impact of the different learning algorithms, operator sets and their combinations. An analysis of the percentage of correctly-classified instances with respect to the algorithms shows no statistically-significant differences between C4.5, DT, RF and SMO for the activity “go to bed”, only VP being perceptibly worse ($F_{4,150}=15.64$, p<0.000). This behavior is similar for the activity “use the toilet” ($F_{4,150}=8.935$, p<0.000), although in this case, RF and SMO present a better average value. The five supervised learning algorithms have the highest difference with respect to correctly-classified instances in the case of the activity “prepare dinner” ($F_{4,150}=98.93$, p<0.000). SMO obtains the highest average value, followed by RF, C4.5, VP and, finally, DT. Globally, the results reveal that SMO and RF get the best percentage of correctly-classified instances ($F_{4,460}=18.92$, p<0.000) with no statistically-significant differences between them. Figure 4 illustrates these results.

**Figure 5:**
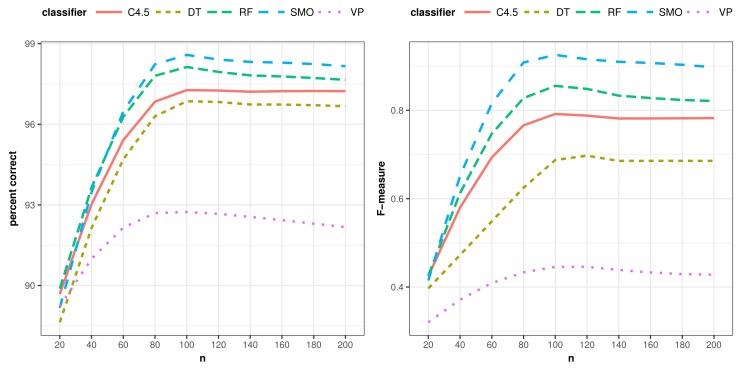
Evolution of the average percentage of correctly-classified instances and F-measure.

In terms of the *F-measure*, all classifiers attain the same positioning, while SMO is slightly better than RF, and the differences among all classifiers are higher ($F_{4,460}=25.05$, p<0.000). Table 9 presents the Duncan test’s groups and differences among elements in both measures. Likewise, Figure 5 shows the average evolution of correctly-classified instances and *F-measure* for each classifier. It is worth mentioning that the improvements in results are not due to the learning algorithms chosen. Their behavior is similar in both the classic and proposed approach. The improvements come from the richer representation of data by the class expressions generated in this work.

No global differences were found when studying the interaction effect between operator sets and algorithms ($F_{12,917}=0.329$, p=0.98), with the only exception of the *classic* approach, which clearly reports lower values for both measures. Table 10 presents such results.

In view of these observations, we can conclude that the most suitable set of operators is S, since there are no significant differences in the results, but nevertheless, it offers a higher performance. In the case of the “go to bed” activity, for example, 3.36 s are needed on an Intel Core i7-6700 (16 GB RAM) to generate and evaluate a hundred features when the S set is used. Instead, 113.77 and 408.78 s are required to produce the same number of features using the sets ACIXMSU and CSM, respectively. The low performance is due to the use of cardinality restrictions in class expressions generated by such operator sets, since their evaluation requires a significant amount of resources. The set CSM is the least efficient because it is the set that generates the most class expressions with cardinality restrictions. An adequate strategy would be to only use the existential quantifier for the generation of class expressions at the beginning of the experiment. This operator produces class expressions that can be evaluated very quickly by the reasoner, which allows us to perform a quick exploratory analysis and validate the design of the ontology. Only from then on is it convenient to test the rest of the DL operators to generate class expressions. The complement, union and intersection operators should be the next to be tested, since the class expressions they generate are also relatively quick to evaluate. The universal quantifier and the cardinality restriction operators are the last ones that should be added to the analysis, since the time required to evaluate the class expressions generated with them requires an extremely high amount of time compared to the former and the improvements are not significant.

Table 11 summarizes the results discussed above. Furthermore, the best results achieved for all the activities in the dataset are shown. The fourth and eighth columns show the improvement of our proposal with respect to the classic approach. The fifth and last columns show the gain achieved by our proposal with respect to the maximum possible gain for each measure. As can be seen, the accuracy of classifiers based on the proposal presented in this paper is improved substantially. The average improvement for all the activities in the dataset is around seventy percent with respect to the maximum possible improvement from the classic approach results. Our proposal only gets slightly worse results with the activity “get drink”, where the classic approach generates a perfect classifier. The difference with respect to our proposal is very small and statistically not significant.

The other two popular activity recognition datasets have been used to test the previous findings. Only the existential quantifier and the SMO algorithm have been used in this case. The second dataset was proposed in [45] and contains a sensor data stream collected at the Washington State University smart apartment. The dataset represents 20 participants performing eight ADL activities in the apartment. The activities were performed individually and sequentially. Each participant performed the same set of activities in any order. This dataset contains 178 activities that are annotated in the stream of state-change sensors generated by 45 sensors. There are eight different activities: answer the phone, choose outfit, clean, fill medication dispenser, prepare birthday card, prepare soup, watch DVD and water plants. The third dataset was proposed in [9] and was collected at the UC Irvine Machine Learning Repository. The dataset represents two participants performing ten ADL activities in their own homes. The activities were performed individually, and this dataset is composed of two instances of data, each one corresponding to a different user and completed in 35 days. In this dataset, the number of sensors is 12, although two of them are never fired in the case of the second participant. In fact, the dataset can be actually considered as two different datasets. Ten activities are annotated in this dataset: breakfast, dinner, leaving, lunch, showering, sleeping, snacking, spare time TV and grooming. Table 12 shows the results obtained for these datasets. As can be seen, our proposal achieves a slightly better average performance for both the percentage of correctly-classified instances and the *F-measure*. There are however activities for which the classic approach performs better than our proposal. In these cases, the loss of performance is more noticeable in the percentage of correctly-classified activities, the differences with respect to the *F-measure* being less noticeable and not statistically significant.

All the results presented above have been obtained when making the size of the temporal windows the same as the average duration of the different types of activities (c=0). To measure the importance of the size of the window in the results, the experiment has been repeated for c=0.25 and c=0.5. However, statistically-significant differences in the results have only been found in the case of the classic approach for the activity “go to bed”, with c=0.5. It goes from a percentage of correctly-classified activities of 91.43% and an *F-measure* of 0.30 to 93.62% and 0.58, respectively. However, it is worth mentioning that increasing the size of the window also increases the number of individuals per activity in the ontology, decreasing the performance of reasoners. For example, the time required to generate and evaluate a hundred features using the set CSM is 1054 seconds for c=0.5, which is more than twice the time required when c=0.

### 4.4. Simulation of a Real Scenario

**Figure 6:**
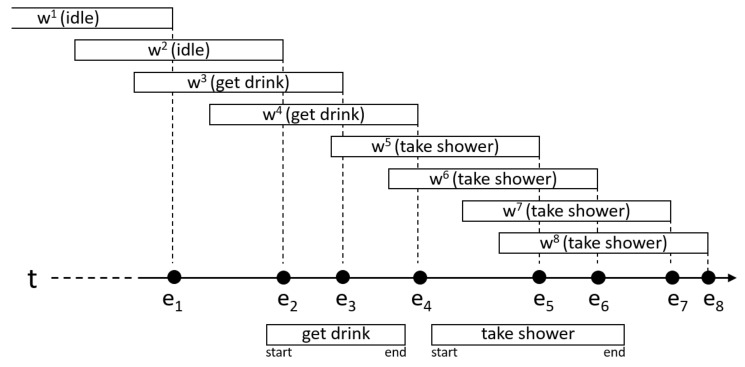
Example of an event-based activity segmentation.

The previous results have been obtained knowing the end of the activity. However, in a real environment, the end label is not known either. To check how the system would behave in a real environment, we have performed another experiment in which we have removed the start and the end labels of the annotated activities. Instead, we have used an event-based segmentation (see Figure 6).

Let $E=\{e_{1},\ldots ,e_{i}\}$ be the set of all the changes produced in the sensors (activations and deactivations). For each of the changes in *E*, a temporal window $w^{i}$ has been generated, whose end must coincide with the instant in which the change eioccurs. The end of the window $w^{3}$ in Figure 6, for example, has been matched to the instant in which the change $e_{3}$ occurs. All the windows have a duration equal to the average duration of the activity being recognized.

An activity $a_{j}$ is being performed during the window $w^{i}$ if they are overlapped for at least a fraction of time *d*. In the experiment, d=0.1, so both intervals must be overlapped at least ten percent of their total duration. The window $w^{2}$ in Figure 6 barely overlaps one of the instances of the activity “get drink” (under the timeline), so it is assumed that no activity was taking place during the window $w^{2}$. Window $w^{5}$ overlaps with instances of two different activities. In these cases, we have chosen to associate the window with the activity that best fits the window. The relative amount of time the intervals are overlapped is used for this. For this reason, the window $w^{5}$ has been assigned to the activity “take shower” and not to the activity “get drink”.

In a real environment, the system would be activated every time a change in a sensor occurs. The objective of the classifier would be to determine if the activity $a_{i}$ is being carried out or has just been carried out at that moment. To evaluate how our proposal would behave in a real environment, we have tried to avoid biases by using two different training and test sets. The windows constructed in the previous section, where we know the end label of each activity, have been used as the training set. The windows generated using the event-based approach, described in this section, have been used as the test set. For simplicity, only the SMO algorithm has been employed. It is the algorithm with the best performance in the previous experiment. The same test and training sets that have been used to evaluate our proposal have been also used to test the classic approach.

The number of individuals and relations in the ontology has been significantly increased; particularly in the case of longer activities, since each window now includes many more sensor changes. For this reason, only the existential quantifier has been used to generate the features. Up to sixty features (n=60) have been generated for each activity. It only takes twenty seconds to evaluate the 2630 windows for most activities. However, in the case of “go to bed” and “leave the house” activities, which are by far the longest, the time required is around one minute. The results are shown in Table 13.

As can be seen, the proposal presented in this paper obtains better results than the classic approach for most of the activities. Only in the case of the activity “prepare dinner” does our proposal have lower accuracy. It is worth mentioning that the activity “prepare dinner” is the activity with less instances in the dataset and the activity with the highest average number of sensor changes. The event-based segmentation has probably broke many of the sequences of changes in different windows and thus the degradation of our proposal, since the generated class expressions mainly describe sequences of changes.

Kasteren et al. [23] reported an average class accuracy of 73.3% for online activity recognition for the same dataset, while our proposal correctly classified 85.25% of the instances. However, it is important to note that they used a very different approach for building the classification models and for the evaluation of the experiments. They used a time-based approach for the segmentation of the activities. The entire dataset was divided into a set of equally-spaced windows of sixty seconds in length. Furthermore, they employed leaving one day out for the evaluation of the experiments. One full day of sensor readings was used for testing, and the remaining days were used for training. Furthermore, there is no information about the precision nor recall, so we can only compare the results by the average percentage of correctly-classified windows and not by the *F-measure*, which better represents the performance of the classifiers.

## 5. Conclusions and Future Work

In this work, we have presented our hybrid methodology for online activity recognition. Unlike offline recognition, in this case, the instant at which the activities are initiated is unknown and, therefore, its duration. To determine the relevant information for each activity instance, temporal windows have been used, the size of which has been determined dynamically, according to the characteristics of each type of activity in the dataset.

Our proposal is based on the use of ontologies for the automatic generation of features vectors, which are used later by supervised learning algorithms to generate the classification models. The features are actually class expressions that have been generated by combining the entities of the ontology in which the dataset is described. The type and complexity of class expressions depend on the DL operators that are used to combine the concepts and properties.

To evaluate the proposal, several experiments have been carried out with the activities of a well-known dataset. In this experiment, the impact of several parameters of the methodology has been measured, such as the set of DL operators employed, the size of the temporal window, the supervised learning algorithm being used to generate the classification model or the number of features generated. The results have been compared with those obtained by the classic approach, in which each feature represents the activation, or not, of each of the sensors in the temporal windows.

The results show an important improvement in the accuracy of the classifiers generated by applying the methodology proposed in this paper. It is only necessary to generate around sixty features to obtain very significant improvements, independent of the set of DL operators used to generate them. In fact, the experimental data reveal that there is no significant difference in the accuracy achieved with the three sets of DL operators used. However, there is much difference in the time required to obtain the results, the set that only uses the existential quantifier clearly requiring less time. No significant differences were observed among the results obtained by the algorithms C4.5, Sequential Minimal Optimization (SMO), random forest and decision table, although SMO obtained slightly better average results. The voted perceptron algorithm is clearly the least suitable for this task.

Our future work is focused on the development of a more suitable model to determine the optimal size and shape of the temporal windows. An adequate design of this model may considerably reduce the amount of resources needed to apply the methodology proposed in this work and facilitate its use in embedded systems.

## Figures and Tables

**Table 1:** Semantics of the OWL logical operators. DL, Description Logic.

	DL Syntax	Manchester Syntax	Semantics
I	$C_{1}⊓C_{2}$	$C_{1}\;and\;C_{2}$	${\left(C_{1}⊓C_{2}\right)}^{I}=\left(C_{1}^{I}\cap C_{2}^{I}\right)$
U	$C_{1}⊔C_{2}$	$C_{1}\;or\;C_{2}$	${\left(C_{1}\cup C_{2}\right)}^{I}=\left(C_{1}^{I}\cup C_{2}^{I}\right)$
C	¬C	notC	${\left(\neg C\right)}^{I}=\Delta_{I}\setminus C^{I}$
S	∃R.C	RsomeC	${\left(\exists R.C\right)}^{I}=\left\{x|\exists y.\langle x,y\rangle \in R^{I}\wedge y\in C^{I}\right\}$
A	∀R.C	RonlyC	${\left(\forall R.C\right)}^{I}=\left\{x|\forall y.\langle x,y\rangle \in R^{I}\to y\in C^{I}\right\}$
X	≤nR.C	RmaxnC	${\left(\geq nR.C\right)}^{I}=\left\{x|card\;\left\{y.\langle x,y\rangle \in R^{I}\wedge y\in C^{I}\right\}\leq n\right\}$
M	≥nR.C	RminnC	${\left(\leq nR.C\right)}^{I}=\left\{x|card\;\left\{y.\langle x,y\rangle \in R^{I}\wedge y\in C^{I}\right\}\geq n\right\}$

**Table 2:** Example of the resultant data.

	*startsWith some*	*hasItem min 2*	
Activity	*Hall-Bedroom_Door_Set*	*Hall-Bedroom_Door_Set*	Positive
1	1	0	1
2	0	1	0
3	1	1	1
4	0	0	1

**Table 3:** Instances per activity in the dataset.

Activity	Instances	Duration (average)	Duration (sd)
Get drink	20	53.25	68.81
Go to bed	24	29,141.63	10,913.63
Leave the house	34	39,823.09	42,045.64
Prepare breakfast	20	202.75	153.61
Prepare dinner	10	2054.00	1185.22
Take a shower	23	573.39	158.53
Use the toilet	114	104.62	101.01
	245		

**Table 4:** Sensor events per activity in the dataset.

Activity	Sensor Events	Sensor Events (average)	Sensor Events (sd)
Get drink	69	3.45	1.00
Go to bed	74	3.08	1.06
Leave the house	113	3.32	2.06
Prepare breakfast	100	5.00	1.30
Prepare dinner	64	6.40	1.58
Take a shower	53	2.30	0.56
Use the toilet	376	3.30	0.81
	1319		

**Table 5:** Performance of the classic approach when using the mean duration of activities as the lengths of temporal windows (c=0). SMO, Sequential Minimal Optimization; VP, Voted Perceptron; DT, Decision Table.

	Percent Correct						F-Measure					
Activity	C4.5	SMO	VP	DT	RF	Best	C4.5	SMO	VP	DT	RF	Best
Get drink	96.86	99.32	95.37	96.87	100.00	100.00	0.83	0.96	0.57	0.80	1.00	1.00
Go to bed	89.26	89.54	90.36	90.23	91.43	91.43	0.00	0.00	0.03	0.00	0.30	0.30
Leave the house	97.96	97.96	94.56	97.02	97.42	97.96	0.94	0.94	0.74	0.91	0.92	0.94
Prepare breakfast	96.19	95.91	94.82	91.02	97.82	97.82	0.75	0.71	0.56	0.29	0.83	0.83
Prepare dinner	95.51	96.18	96.06	94.39	97.82	97.82	0.42	0.51	0.30	0.33	0.64	0.64
Take a shower	98.36	98.23	94.04	90.63	98.63	98.63	0.90	0.89	0.44	0.00	0.91	0.91
Use the toilet	89.26	88.19	84.91	87.08	91.44	91.44	0.89	0.87	0.84	0.88	0.91	0.91

**Table 6:** “Go to bed” classification performance.

		Percent Correct						F-Measure					
	$|F^{k}|$	C4.5	SMO	VP	DT	RF	Best	C4.5	SMO	VP	DT	RF	Best
Classic	14	89.26	89.54	90.36	90.23	91.43	**91.43**	0.00	0.00	0.03	0.00	0.30	**0.30**
ACIXMSU	20	90.23	90.23	90.23	90.23	89.69	90.23	0.00	0.00	0.00	0.00	0.00	0.00
ACIXMSU	40	90.23	89.70	90.23	90.23	89.14	90.23	0.00	0.00	0.00	0.00	0.00	0.00
ACIXMSU	60	91.29	93.46	90.23	90.23	93.33	93.46	0.45	0.57	0.00	0.00	0.50	0.57
ACIXMSU	80	*98.78*	*98.64*	92.55	*98.78*	*98.51*	**98.78**	*0.92*	*0.91*	0.29	0.92	0.91	**0.92**
ACIXMSU	100	*98.78*	*98.78*	91.06	*98.78*	*98.37*	98.78	*0.92*	*0.92*	0.14	*0.92*	*0.90*	0.92
CSM	20	90.23	90.23	90.23	90.23	89.69	90.23	0.00	0.00	0.00	0.00	0.00	0.00
CSM	40	90.23	89.70	90.23	90.23	89.14	90.23	0.00	0.00	0.00	0.00	0.00	0.00
CSM	60	91.29	93.46	90.23	90.23	93.33	93.46	0.45	0.57	0.00	0.00	0.50	0.57
CSM	80	*98.78*	*98.78*	93.36	*98.78*	*98.51*	**98.78**	*0.92*	*0.92*	0.40	*0.92*	*0.91*	**0.92**
CSM	100	*98.78*	*98.78*	93.22	*98.78*	*98.51*	98.78	*0.92*	*0.92*	0.35	*0.92*	*0.91*	0.92
S	20	90.23	90.23	90.23	90.23	90.23	90.23	0.00	0.00	0.00	0.00	0.00	0.00
S	40	*98.78*	*98.78*	92.27	*98.78*	*98.64*	**98.78**	*0.92*	*0.92*	0.24	*0.92*	*0.91*	**0.92**
S	60	*98.78*	*98.23*	91.73	*98.78*	*98.37*	98.78	*0.92*	*0.89*	0.22	*0.92*	*0.89*	0.92
S	80	*98.78*	*97.97*	90.51	*98.78*	*98.37*	98.78	*0.92*	*0.87*	0.04	*0.92*	*0.89*	0.92
S	100	*98.78*	*97.97*	90.91	*98.78*	*98.09*	98.78	*0.92*	*0.87*	0.09	*0.92*	*0.87*	0.92

**Table 7:** “Use the toilet” classification performance.

		Percent Correct						F-Measure					
	$|F^{k}|$	C4.5	SMO	VP	DT	RF	Best	C4.5	SMO	VP	DT	RF	Best
Classic	14	89.26	88.19	84.91	87.08	91.44	**91.44**	0.89	0.87	0.84	0.88	0.91	**0.91**
ACIXMSU	20	80.94	*80.12*	79.71	*76.72*	*81.76*	81.76	*0.79*	*0.78*	0.78	*0.76*	*0.80*	0.80
ACIXMSU	40	84.82	83.99	84.12	82.90	85.89	85.89	0.85	0.84	0.84	0.83	0.86	0.86
ACIXMSU	60	*95.68*	*96.77*	89.73	*94.72*	*96.49*	96.77	*0.95*	*0.97*	0.90	*0.94*	*0.96*	0.97
ACIXMSU	80	*94.88*	*97.72*	89.29	*94.99*	*97.03*	**97.72**	0.95	*0.98*	0.89	*0.94*	*0.97*	**0.98**
ACIXMSU	100	*94.88*	*97.18*	89.56	*94.99*	*96.89*	97.18	0.95	*0.97*	0.89	*0.94*	*0.97*	0.97
CSM	20	80.94	*80.12*	79.71	*76.72*	*81.76*	81.76	*0.79*	*0.78*	0.78	*0.76*	*0.80*	0.80
CSM	40	84.82	83.99	84.12	82.90	85.89	85.89	0.85	0.84	0.84	0.83	0.86	0.86
CSM	60	*95.68*	*96.77*	89.73	*94.72*	*96.49*	96.77	*0.95*	*0.97*	0.90	*0.94*	*0.96*	0.97
CSM	80	*94.88*	*97.72*	90.26	*94.99*	*97.57*	97.72	0.95	*0.98*	0.90	*0.95*	*0.97*	**0.98**
CSM	100	*94.88*	*97.86*	88.32	*94.86*	*97.02*	**97.86**	0.95	*0.98*	0.88	*0.94*	*0.97*	0.98
S	20	*80.86*	*79.52*	81.81	80.44	*84.77*	84.77	*0.80*	*0.79*	0.81	0.80	0.85	0.85
S	40	*95.54*	*97.04*	91.06	*94.59*	*96.90*	97.04	0.95	*0.97*	0.91	*0.94*	*0.97*	0.97
S	60	*95.54*	*97.71*	89.31	*94.99*	*96.76*	**97.71**	*0.95*	*0.98*	0.89	*0.94*	*0.97*	**0.98**
S	80	*95.54*	*97.30*	88.47	*94.99*	*97.02*	97.30	*0.95*	*0.97*	0.89	*0.94*	*0.97*	0.97
S	100	*95.54*	*97.31*	88.73	*94.72*	*96.48*	97.31	*0.95*	*0.97*	0.88	*0.94*	*0.96*	0.97

**Table 8:** “Prepare dinner” classification performance.

		Percent Correct						F-Measure					
	$|F^{k}|$	C4.5	SMO	VP	DT	RF	Best	C4.5	SMO	VP	DT	RF	Best
Classic	14	95.51	96.18	96.06	94.39	97.82	**97.82**	0.42	0.51	0.30	0.33	0.64	**0.64**
ACIXMSU	20	97.96	97.83	96.33	*97.96*	97.42	97.96	0.50	0.49	0.13	0.50	0.47	0.50
ACIXMSU	40	97.56	98.79	96.47	96.06	98.78	98.79	0.49	0.77	0.13	0.12	0.72	0.77
ACIXMSU	60	97.28	99.06	96.87	96.20	98.51	**99.06**	0.46	*0.88*	0.23	0.16	0.63	**0.88**
ACIXMSU	80	97.28	98.92	96.73	96.20	98.65	98.92	0.46	0.84	0.20	0.16	0.69	0.84
ACIXMSU	100	97.28	98.92	96.18	96.20	98.37	98.92	0.46	0.80	0.10	0.16	0.62	0.80
CSM	20	97.96	97.83	96.33	*97.96*	97.42	97.96	0.50	0.49	0.13	0.50	0.47	0.50
CSM	40	97.56	98.79	96.47	96.06	98.78	98.79	0.49	0.77	0.13	0.12	0.72	0.77
CSM	60	97.28	99.06	96.87	96.20	98.51	**99.06**	0.46	*0.88*	0.23	0.16	0.63	**0.88**
CSM	80	97.28	99.06	96.59	96.20	98.66	99.06	0.46	*0.88*	0.17	0.16	0.69	0.88
CSM	100	97.28	98.92	96.46	96.20	98.91	98.92	0.46	0.84	0.13	0.16	0.76	0.84
S	20	97.29	96.34	96.33	97.43	96.07	97.43	0.48	0.36	0.24	0.48	0.39	0.48
S	40	97.69	98.37	97.01	96.61	98.36	98.37	0.60	0.74	0.27	0.26	0.60	0.74
S	60	97.69	98.92	96.86	96.61	97.95	98.92	0.60	0.85	0.31	0.26	0.57	0.85
S	80	97.69	*99.59*	96.73	96.61	98.36	**99.59**	0.60	*0.97*	0.20	0.26	0.69	**0.97**
S	100	97.28	*99.45*	97.69	96.48	98.37	99.45	0.50	0.89	0.43	0.26	0.62	0.89

**Table 9:** Duncan test’s groups of classifiers by the percentage of correctly-classified instances.

Measure	Group	Classifier	Mean	SD
Precision	a	SMO	96.581	4.395
	a	RF	96.390	3.759
	ab	C4.5	95.708	4.006
	b	DT	95.087	4.444
	c	VP	91.958	4.161
F-measure	a	SMO	0.815	0.246
	ab	RF	0.758	0.244
	b	C4.5	0.708	0.283
	c	DT	0.608	0.378
	d	VP	0.414	0.034

**Table 10:** Duncan test’s groups of datasets by the percentage of correctly-classified instances.

Measure	Group	DL Operators	Mean	SD
Precision	a	S	96.662	4.095
	a	ACIXMSU	95.117	4.574
	a	CSM	95.025	4.641
	b	classic	91.444	3.758
F-measure	a	S	0.697	0.308
	a	ACIXMSU	0.655	0.335
	a	CSM	0.647	0.345
	b	classic	0.461	0.359

**Table 11:** Global classification performance.

	Percent Correct				F-Measure			
Activity	Classic	Proposal	Gain	% Max.	Classic	Proposal	Gain	% Max.
				Gain				Gain
Go to bed	91.43	98.78	7.35	86%	0.30	0.92	0.62	89%
Prepare dinner	97.82	99.59	1.77	81%	0.83	0.97	0.14	82%
Use the toilet	91.44	97.86	6.42	75%	0.91	0.98	0.07	78%
Get drink	100.00	99.59	−0.41	-	1.00	0.97	−0.03	-
Leave the house	97.96	100.00	2.04	100%	0.94	1.00	0.06	100%
Take a shower	98.63	98.77	0.14	10%	0.91	0.93	0.02	22%
Prepare breakfast	97.82	98.78	0.96	44%	0.83	0.92	0.09	53%
	96.44	99.07	2.63	66%	0.79	0.96	0.17	72%

**Table 12:** Global classification performance on the Singla and Ordoñez datasets.

		Percent Correct			F-Measure		
Dataset	Activity	Classic	Proposal	Gain	Classic	Proposal	Gain
Singla	Answer the phone	98.22	98.81	0.59	0.89	0.94	0.05
Singla	Choose outfit	100.00	100.00	0.00	1.00	1.00	0.00
Singla	Clean	98.81	98.80	−0.01	0.95	0.95	0.00
Singla	Fill medication dispenser	99.02	97.81	−1.21	0.94	0.89	−0.05
Singla	Prepare birthday card	100.00	98.39	−1.61	1.00	0.93	−0.07
Singla	Prepare soup	100.00	100.00	0.00	1.00	1.00	0.00
Singla	Wash DVD	98.60	100.00	1.40	0.95	1.00	0.05
Singla	Water plants	98.60	98.80	0.20	0.94	0.95	0.01
Ordoñez (a)	Breakfast	99.55	99.55	0.00	0.90	0.94	0.04
Ordoñez (a)	Grooming	96.53	97.12	0.59	0.92	0.94	0.02
Ordoñez (a)	Leaving	99.55	100.00	0.45	0.98	1.00	0.02
Ordoñez (a)	Lunch	99.50	100.00	0.50	0.88	0.90	0.02
Ordoñez (a)	Showering	100.00	100.00	0.00	1.00	1.00	0.00
Ordoñez (a)	Sleeping	100.00	100.00	0.00	1.00	1.00	0.00
Ordoñez (a)	Snack	100.00	99.70	−0.30	1.00	0.98	−0.02
Ordoñez (a)	Spare_Time_TV	98.49	100.00	1.51	0.98	1.00	0.02
Ordoñez (a)	Toileting	97.29	97.29	0.00	0.86	0.86	0.00
Ordoñez (b)	Breakfast	96.06	96.80	0.74	0.41	0.62	0.21
Ordoñez (b)	Dinner	97.55	97.62	0.07	0.00	0.33	0.33
Ordoñez (b)	Grooming	93.60	92.71	−0.89	0.86	0.83	−0.03
Ordoñez (b)	Leaving	95.38	97.70	2.32	0.71	0.86	0.15
Ordoñez (b)	Lunch	97.10	97.10	0.00	0.00	0.42	0.42
Ordoñez (b)	Showering	100.00	100.00	0.00	0.60	0.60	0.00
Ordoñez (b)	Sleeping	94.05	100.00	5.95	0.34	1.00	0.66
Ordoñez (b)	Snack	90.93	91.97	1.04	0.40	0.60	0.20
Ordoñez (b)	Spare_Time_TV	88.55	92.35	3.80	0.73	0.85	0.12
Ordoñez (b)	Toileting	93.08	94.57	1.49	0.80	0.85	0.05
Average		97.42	98.04	0.62	0.78	0.86	0.08

**Table 13:** Global classification performance in a simulation of a real scenario.

	Percent Correct		F-Measure	
Activity	Classic	Proposal	Classic	Proposal
Go to bed	56.91	60.16	0.00	0.20
Prepare dinner	90.46	96.29	0.51	0.39
Use the toilet	84.84	80.16	0.76	0.82
Get drink	92.54	92.30	0.52	0.52
Leave the house	76.39	78.56	0.82	0.83
Take a shower	92.30	94.13	0.41	0.44
Prepare breakfast	93.86	95.13	0.36	0.50
	83.90	85.25	0.48	0.53
